# Immunosuppressive Protein Signatures Carried by Syncytiotrophoblast-Derived Exosomes and Their Role in Human Pregnancy

**DOI:** 10.3389/fimmu.2021.717884

**Published:** 2021-07-26

**Authors:** Lucia Mincheva-Nilsson

**Affiliations:** Section of Infection and Immunology, Department of Clinical Microbiology, Faculty of Medicine, Umeå University, Umeå, Sweden

**Keywords:** exosomes, immune suppression, maternal-fetal tolerance, human placenta, pregnancy

## Abstract

The syncytiotrophoblast (STB) of human placenta constitutively and throughout pregnancy produces and secretes exosomes - nanometer-sized membrane-bound extracellular vesicles from the endosomal compartment that convey cell-cell contact ‘by proxy’ transporting information between donor and recipient cells locally and at a distance. Released in the maternal blood, STB-derived exosomes build an exosomal gradient around the feto-placental unit acting as a shield that protects the fetus from maternal immune attack. They carry signal molecules and ligands that comprise distinct immunosuppressive protein signatures which interfere with maternal immune mechanisms, potentially dangerous for the ongoing pregnancy. We discuss three immunosuppressive signatures carried by STB exosomes and their role in three important immune mechanisms 1) NKG2D receptor–mediated cytotoxicity, 2) apoptosis of activated immune cells and 3) PD-1-mediated immunosuppression and priming of T regulatory cells. A schematic presentation is given on how these immunosuppressive protein signatures, delivered by STB exosomes, modulate the maternal immune system and contribute to the development of maternal-fetal tolerance.

## Introduction

Considering the implantation and development of a semiallogeneic fetal allograft in the uterine cavity, it is obvious that mammalian pregnancy defies the laws of transplantation. The peaceful co-existence between mother and fetus during pregnancy was defined by the Nobel Laureate Sir Peter Medawar as “a paradox of Nature”. His seminal work (1) has been an inspiration for scientists in many years and has been instrumental for the research progress in reproductive immunology. A multitude of mechanisms, hormones and signal substances (2, 3) has been proven to participate in the immune regulation during pregnancy. Despite many achievements in understanding the transient maternal-fetal tolerance, the overall solution of the feto-maternal immunologic challenge still remains elusive.

The successful outcome of mammalian pregnancy is dependent on the key organ of mammalian reproduction – the placenta. The human hemochorial placenta mediates not only hormonal, nutritional and oxygen support to the developing fetus but also plays a decisive role as a supplier of steroid- and protein pregnancy hormones and an immunomodulatory organ, molding the maternal immune system towards acceptance and protection of pregnancy. All these functions are effectuated by a specific and unique cell type, the syncytiotrophoblast (STB), a continuous plasma membrane-bound layer of multinucleated cytoplasm that completely covers the multitudinous chorion villi and comprises about 70% of all cells in the placenta (4, 5). In addition, STB constitutively produces extracellular vesicles (EV) of different biogenesis, morphology and size which are secreted into the maternal circulation and participate in the maternal-fetal cross-talk during pregnancy. Following the latest position statement of the International Society for Extracellular Vesicles MISEV 2018 (6) the three main types of STB EV should be denominated as STB-derived, small (<200nm) CD63+/CD81+, medium and large (>200 nm), annexin V-staining+ vesicles. However, according to MISEV 2018, if imagining techniques, such as electron microscopy (EM), immunoelectron microscopy (IEM) or live imagining, are used to study their formation and release, the nomenclatures shedding microvesicles (in placenta STB microparticles, STBM), apoptotic bodies/blebs and exosomes may be used. The vast majority of the original reports, used in this review, are based on solid isolation procedures and biogenesis and release studies using imagining techniques, thus, the STB-derived small, medium and large EVs will be denominated as exosomes, STBM, and apoptotic bodies, respectively.

**Figure 1:**
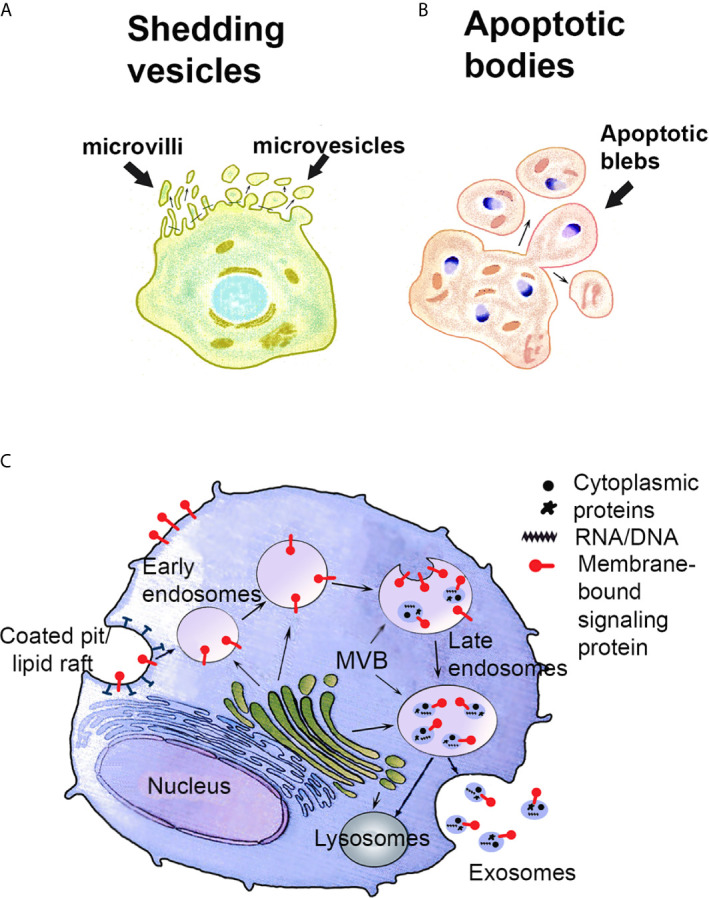
Schematic presentation of the biogenesis of shedding vesicles **(A)**, apoptotic bodies **(B)** and exosomes **(C)**. **(A, B)** Shedding vesicles such as STBM and shed microvilli and apoptotic bodies are produced by blebbing of the plasma membrane. **(C)** Recycled or newly synthesized proteins are sorted to the limiting membrane of multivesicular bodies (MVB). Exosomes are produced by invagination of the MVB membrane. Exosomes carry on their surface the proteins sorted to the MVB membrane. The cargo inside comprises cytosolic proteins and nucleic acids. Exosome-filled MVB are either exocytotic, i.e. fuse with the plasma membrane and release their content as exosomes to the extracellular space or degradative, sending their content to lysosomes for degradation. Modified from ref. (12).

The free apical surface of the STB-covered placental villi that is in direct contact with the maternal blood, is rich in highly pleomorphic microvilli and surface projections as illustrated by EM (5, 7). Morphological observations show that microvesicles and even whole microvilli are released from the apical syncytial plasma membrane (7, 8). Furthermore, the growth and branching of the placental villi during gestation to increase the overall area of STB, demands a high trophoblast turnover with both enhanced cell differentiation and apoptosis, and thus, a second type of placental vesicles, large apoptotic bodies/blebs are produced by blebbing/fragmentation of dying cells (5, 7, 8). This normal shedding activity and cell death produce shedding vesicles called STB microvesicles/microparticles (STBM) and apoptotic blebs/bodies - vesicles that are forcefully enhanced in numbers in pathological disorders such as spontaneous abortions and pre/eclampsia (9–12). The third and smallest type of STB vesicles are the exosomes (12–14), defined as nanometer-sized, membrane-bound extracellular vesicles of endosomal origin, released into the intercellular space and maternal blood when multivesicular bodies (MVB) fuse their membrane with the plasma membrane of STB. The three main types of extracellular vesicles (14) released from STB are illustrated in **Figure 1**, where also the biogenesis of the exosomes is illustrated (**Figure 1**). Their main physical and biochemical characteristics are summarized in **Table 1**. As can be seen, there is some overlapping in some of the physical and biochemical properties, therefore, a reliable isolation procedure is required to obtain pure populations of EV. The golden standard for exosome isolation is serial centrifugations and ultrafiltration through 200 nm filter for discarding larger EVs and cell debris, followed by a sucrose gradient ultracentrifugation at 100 000 - 110 000xg for 1-3 hours. This method, and post-isolational methods for verifying the exosomal yield are described in detail by our group and can be found in a chapter in Current Protocols in Immunology (15). Immunomodulation of the maternal immune response is a central part of the solution of the immune challenge during mammalian pregnancy and the STB-derived EVs play an important role in the creation of an active immune tolerance at the maternal-fetal interface. This review will focus on the STB-derived exosomes expressing protein signatures that convey immune inhibition.

**Table 1:** Some characteristics of extracellular vesicles produced by human syncytiotrophoblast [modified from ref. (11)].

Characteristics	Exosomes	Shed Microvesicles/Microparticles	Apoptotic blebs/bodies
**Size**	30-150 nm	0.1-2 μm	50 nm – 5 μm
**Density in sucrose **	1.13 – 1.19 g/ml	Undetermined	1.16-1.28 g/ml
**Sedimentation (xg)**	100,000 -110,000	10,000 -100,000	1,500 – 100,000
**Morphological shape in electron microscopy**	Cup shaped, electron translucent	Various shapes, round, elongated and cylinder-like, electron-dense and/or electron translucent	Irregular and heterogeneous in shape
**Lipid membrane composition**	Cholesterol-, shingomyelin-, and ceramid-rich lipid rafts, expose phosphatidylserine	Expose phosphatidylserine, some enriched in cholesterol and diacylglycerol, some undetermined	Undetermined, expose phosphatidylserine
**Specific marker(s) for identification**	Tetraspanins (CD63, CD9, CD81), ESCRT complex members (Alix, TSG101)	Integrins, selectins, CD40* and others, depending on the cell type	Histones, DNA
**Origin in the cell**	Endosomal compartment - multivesicular bodies (MVB)	Plasma membrane	Fragments of dying cells, undetermined
**Mechanism of sorting**	ESCRT-complexes dependent pathway linked to syndecans: syndecan-syntenin-ALIX	Budding from the plasma membrane	Fragments of dying cells, undetermined
	Ceramid-dependent pathway		
	Others suggested: tetraspanins CD9, CD82, CD63;		
	SIMPLE/LITAF		
**Biogenesis**	Inward budding of MVB’s limiting membrane	Fragmentation and detachment of the cytoskeleton, cleavage by activated seine proteases causing destabilization of the plasma membrane	Actin – myosin fibril contraction, release of lactate dehydrogenase
**Intracellular storage**	Yes	No	No
**Mode of release/secretion**	Exocytosis by fusion of MVB with the plasma membrane	Plasma membrane blebbing	Plasma membrane blebbing and cellular fragmentation

*CD40 is not expressed by STBM but is suggested for other shed microvesicles.

## General Characteristics of Syncytiotrophoblast-Derived Placental Exosomes

Exosomes *per se* are defined as membrane-bound extracellular vesicles secreted from the endosomal compartment. They have a distinct detergent-resistant membrane, enriched in tetraspanin-, cholesterol- and sphingolipid molecules. They are cup-shaped, 30-100 nm in size when observed in electron microscopy (EM) and 30-150 nm in size measured by nanoparticle tracking instruments such as ZetaView^®^ and Nanosight^®^. The upper size limit of 100 nm measured by EM is due to shrinkage occurring from fixation for EM analysis. The cup-shape is an artefact due to a collapse in the spherical form of the exosomes, caused by the prolonged ultracentrifugation step in the isolation procedure. Among all extracellular vesicles known so far, exosomes are the only ones that become cup-shaped by this manipulation, probably due to their special, lipid-rich, robust and tough, content-protective exosomal membrane.

STB of human placenta constitutively produces and secretes exosomes. The syncytioplasm has an elaborate system of endosomal membranes and is very rich in MVB, free ribosomes, rough endoplasmic reticulum with dilated cisternae and randomly distributed Golgi complexes suggesting vigorous synthesis of proteins and exosome production and release (4, 5, 7). The exosomal cargo comprises proteins and nucleic acids such as DNA, micro- and lncRNA and reflects the cell from which the exosomes originate. The total cargo per exosome is estimated to ≤ 100 proteins and ≤ 10,000 nucleotides (16). Most/all proteomic- and nucleic acid analyses of isolated exosomes from various sources, report a vastly higher number of proteins and nucleic acids than what can be accommodated in an exosome, indicating that the exosomal cargo is not uniformly distributed but varies in molecule content in each exosome and hence, the proteomic and microarray results represent the overall total cargo, carried by the whole moiety of isolated exosomes, for detailed information on proteomics and nucleic acid studies see ExoCarta (http://www.exocarta.org) (17). We have studied the biogenesis, morphology and release of STB exosomes in early normal pregnancy by EM and IEM (12–14, 18–20) and analyzed their protein content by proteomics. **Table 2** presents a short list of proteins, commonly identified in exosomes, which were also identified in STB-exosomes (13, 14). Placental alkaline phosphatase (PLAP) is constitutively expressed on the surface of the STB exosomes and comprise an “address tag”/marker molecule for identification of human STB-derived exosomes.

**Table 2:** List of proteins, commonly identified in exosomes, revealed in placental exosomes by proteomic analysis [from ref. (11)].

Protein group	Members
Tetraspanins	CD9
	CD63
MVB biogenesis	Ubiquitin
	TSG101
	Alix
	Vacuolar sorting protein 29 (ESCRT)
	Charged MVB proteins 1B and 4B (ESCRT)
Adhesion, targeting	Integrins α5, αV, β1, β3
	CD47
	Transferrin receptor
	Epidermal growth factor receptor
	Liprin b-2
Cytoskeleton proteins	Actin
	Myosin
	Tubulin
	Ezrin
	Profilin 1
	Cofilin 1
Apoptosis regulation	Programmed cell death proteins 6 and 10
Protein biosynthesis and degradation	60S ribosomal proteins
	40S ribosomal proteins
	Elongation factors 1-α1, α2, α3 and γ
	Proteasome α4 subunit
	Proteasome α5 subunit
	Proteasome 26S non-ATPase subunit
Signal transduction	14-3-3 proteins
	Rab 1A, 1B, 35
	Ras-related proteins 1B and R
	Guanine nucleotide binding protein
	Ras GTPase-activating protein
	Transforming protein RhoA
	Sorcin
Enzymes	α-enolase
	5´ nucleotidase
	Dipeptidyl peptidases
Other membrane transport and fusion proteins	Annexins
	Rab proteins: 2A, 5A, 5B, 5C, 6, 7, 10, 14
	Clatrin heavy chain
	Copine-3
	Dysferlin
	Testilin
	Myoferlin
	Syntaxin
	Vesicle transport through interaction protein 1B
Others	Histones
	LAMP2 (CD107B)
	Multidrug resistence protein 1
	S-100 proteins
	Lysosomal membrane protein 2
	Protein DI-1

ESCRT, Endosomal-sorting complexes required for transport; MVB, Multivesicular body.

A great variety of biological functions have been ascribed to exosomes – from a role in embryogenesis, differentiation and development to other biological and immunological functions such as intercellular signaling and cell-cell communication by proxy, delivery and internalization of proteins to plasma membranes and cytoplasm of recipient cells, pro- and anti-apoptosis, antigen presentation, immune regulation, reprogramming of recipient cells by exosomal delivery of bioactive nucleic acids such as DNA, mRNA, micro- and lncRNA (21–26). Exosomes are able to enhance or inhibit the immune response and thus could be either immunoactivating or immunosuppressive/inhibitory. In general, exosomes, produced by immune cells with antigen presenting function (APC), such as macrophages/monocytes, dendritic cells and B cells, carry MHC molecules and can activate other immune cells either directly acting as miniature antigen presenters by proxy, or indirectly, by being uptaken and their cargo processed and presented on the MHC class II molecules by APC that activate the T helper cells and eventually lead to induction or boostering of various immune responses like cytokine production, cytotoxicity, antibody production and priming of effector T cells (12, 13, 21). In contrast, the majority of exosomes produced by epithelial cells from various organs and tumors are immunosuppressive. In health, normal epithelium-derived exosomes, produced at a low rate, are immunosuppressive and promote homeostasis thus keeping the immune system at bay (27). STB-derived exosomes and STBM have a variety of functions, both stimulatory and inhibitory as reviewed in (28). Placental exosomes, internalized by monocytes were shown promote monocyte migration and production of proinflammatory cytokines (29). STBM were found to activate neutrophils and induce formation of NETS, a function that was enhanced in STBM from pre-eclamptic placentas (30). These functions might be associated with infection protection of the feto-maternal unit. Various cancers constitutively secrete high amounts of immunosuppressive exosomes that “highjack” and dysregulate normal immune responses to promote tumor establishment, growth and metastasis (31, 32). The majority of STB-derived exosomes express a distinct set of immune molecules/ligands comprising immunosuppressive protein signatures which interfere with the maternal immune system in a similar way as tumor exosomes do with the host immune system. Below, the immunosuppressive protein signatures of STB exosomes and their effect on three immune mechanisms will be discussed.

## NKG2D Ligand-Expressing STB Exosomes Suppress the Maternal Cytotoxic Response Towards the Semiallogeneic Fetus

The placental villi are directly exposed to the maternal blood and could be subjected to a cytotoxic maternal attack destroying the STB layer and disturbing the vital placental function. The STB is devoid of classical MHC class I and II molecules and thus protected from T cell –mediated killing, but the lack/truncated expression of MHC can instead activate the maternal NK cells. The solution of this dilemma is the constitutive secretion of STB exosomes that carry the non-classical MHC-related proteins MICA/B and UL-16 binding proteins 1 to 5 (ULBP1-5), ligands of the major activating NK-cell receptor NKG2D (12, 13, 18, 19). The two families of NKG2D ligands, MICA/B and ULBP are differentially expressed by STB – while MICA/B are both expressed on the STB membrane and on the endosomal membrane of MVB where MICA/B exosomes are produced, ULBP 1-5 are solely expressed in the endosomal compartment and secreted by exosomes (18, 19) suggesting that MICA/B expression can be present on the membrane of STBM. This indicates that in pregnancy conditions where inflammation is a dominant event, such as pre-eclampsia, the MICA/B expression on the STB membrane together with the intensified STBM secretion plays a central role in the destruction of the placental villi leading to miscarriage (11, 12). STBM production, as well as exosome production, is a physiological process in normal pregnancy and the normal balance between inflammation and suppression is slightly tilted towards a light inflammation that could be beneficial both for infection protection during pregnancy and also for upregulation (20) of the exosome production and secretion as reviewed in (12). We were first to report that interaction of NKG2D ligand-bearing placental exosomes with the NKG2D receptor causes a selective and dose-dependent downregulation of the receptor on NK-, CD8^+^T- and γδT cells. The “decoy” action of these exosomes only internalizes the cognate receptor leaving the perforin-dependent lytic machinery intact. The cells are therefore able to restore the receptor expression and the cytotoxic potency when they re-enter the peripheral blood circulation, away from the feto-maternal unit (18, 19). This sophisticated modulation of the NKG2D-mediated killing ensures the protection of the fetus from maternal cytotoxic attack.

## FasL- and Trail-Expressing STB Exosomes Induce Apoptosis in Activated Immune Cells thus Maintaining the Immunologic Privilege of the Fetus

**Figure 2:**
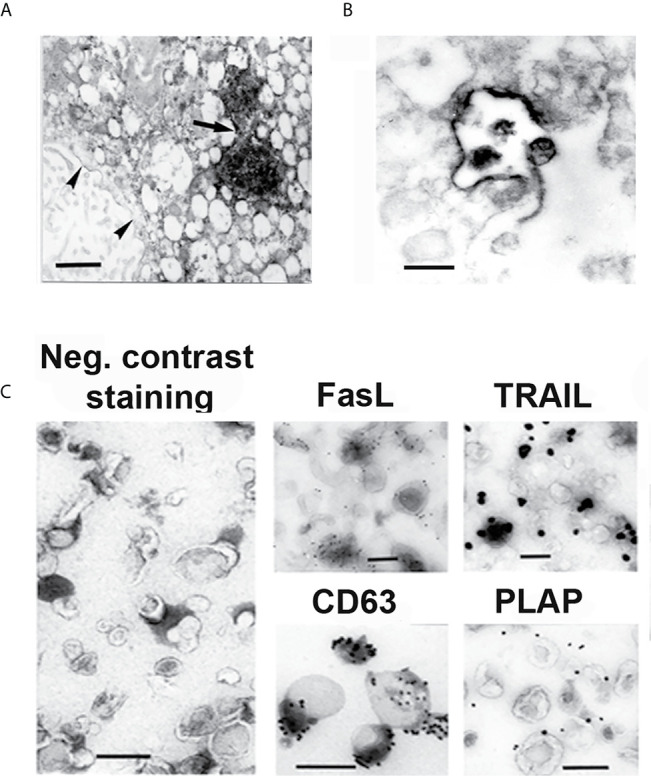
EM and IEM micrographs showing IHC- **(A, B)** and immunogold **(C)** staining of syncytiotrophoblast and isolated exosomes. **(A)** Syncytiotrophoblast stained for FasL. **(B)** Exocytotic MVB, stained for TRAIL, opens up and releases exosomes into the extracellular space. **(C)** Isolated STB exosomes, stained by negative contrast, showing their morphology, and stained by immunogold, showing FasL and TRAIL expression on their surface. Anti-CD63 staining identifies that the isolated extracellular vesicles are exosomes, and anti-placental alkaline phosphatase (PLAP) staining verifies that the exosomes have STB origin. Arrow points at two MVB filled with FasL stained exosomes. Arrowheads point at FasL stained perinuclear membranes. Bars represent 100 nm. Reproduced with copyright permission from ref. (41).

Apoptosis is one of the proposed mechanisms of tolerance in implantation and pregnancy (33). Apoptosis is detected in placenta throughout pregnancy and has been shown to participate in trophoblast invasion, differentiation and turnover during placental formation (34), and in the creation of maternal immune tolerance towards the fetus (35–37). FasL and TRAIL are constitutively expressed in human placenta (38–40). Our morphologic studies reveal that their expression is restricted to the MVBs of STB where these molecules are released on exosomes as illustrated in **Figure 2** (14, 41). Furthermore, these molecules are expressed on the exosomal surface as oligomerized hexamers ready to form DISC complexes and initiate apoptosis (41). Functional *in vitro* studies show that the FasL and TRAIL-bearing STB exosomes induce apoptosis in activated lymphocytes in a dose-dependent manner (41, 42). The physiological importance of these molecules in pregnancy is associated with the immune privilege of the fetus, and is governed by mechanisms similar to those in other immune privileged sites, the eye being the site more thoroughly studied (43–45). Activated maternal lymphocytes, potentially dangerous to the fetus, are eliminated by apoptosis. The non-inflammatory apoptotic cell-death induction, associated with immune privilege, is only possible if expression and aggregation of these pro-apoptotic molecules is provided on the exosomal membrane, avoiding FasL and TRAIL expression on the cellular membrane that either provokes inflammation or rejection; or generates soluble molecules, able to block apoptosis (46–48). Thus, the apoptotic activity, necessary for the maintenance of the immune privilege of the fetus is linked to the placenta’s exosome-secreting ability.

## PD-1 Ligand-Carrying STB-Derived Exosomes Participate in Immune Checkpoint Interactions in Pregnancy

Activation of the immune system to defend against harmful antigens is accompanied by inflammation and tissue damage. To minimize these side effects, immune checkpoint molecules, such as CTLA-4, TIM-3 and PD-1, are negative regulators of the immune response. They exhaust effector cells and enhance T regulatory cell and thereby promote tolerance and tissue homeostasis. The immunological challenge of mammalian pregnancy evokes the question for a possible role of immune checkpoint molecules in the maternal immune tolerance towards the fetus. CTLA-4, TIM-3 and PD-1 have been studied in pregnancy by different *in vivo* and *in vitro* approaches in animal and human experiments as reviewed in (49). The role of STB exosomes in PD-1/PD-L1 checkpoint pathway has been extensively studied (49–53). PD-1/B7 is a transmembranal receptor on T-, B-, NK- and antigen presenting cells. Upon binding to its ligands PD-L1/B7-H1 and PD-L2/B7-H2, expressed on immune cells and tissues, such as placenta, heart, and spleen, a strong inhibitory signal is generated downregulating effector cell activation. PD-L1/B7-H1 and PD-L2/B7-H2 ligands are upregulated by inflammatory cytokines and INF-γ (49). PD-L1/B7-H1 is differentially expressed by extravillous- and syncytiotrophoblast throughout pregnancy (50–52). While the PD-L1/B7-H1 expression on extravillous trophoblast diminish, the STB expression enhances to higher and higher levels as the pregnancy progresses. It is likely regulated by local oxygen tension and cytokines such as IFN-γ (50–52). Solid evidence shows that the PD-L1 and 2 are processed through the endosomal compartment and released from the placenta *via* exosomes (50–53) thus expression of PD-L1 and 2 on other EV, such as STBM, would be unlikely, and if shown, might be due to contamination in the isolation procedure. The suggested role of PD-L1 and 2-carrying exosomes (50–53) is to downregulate/exhaust maternal immune effector cells potentially dangerous to the fetus and to prime naïve cells to T regulatory cells (Treg) by binding to the cognate PD-1 receptor on maternal immune cells. In support of the latter, there are several studies reporting enhanced numbers of Treg cells in human (54–57) and murine (58) normal pregnancy decidua, their role being to promote maternal-fetal tolerance.

## Discussion and Concluding Remarks

**Figure 3:**
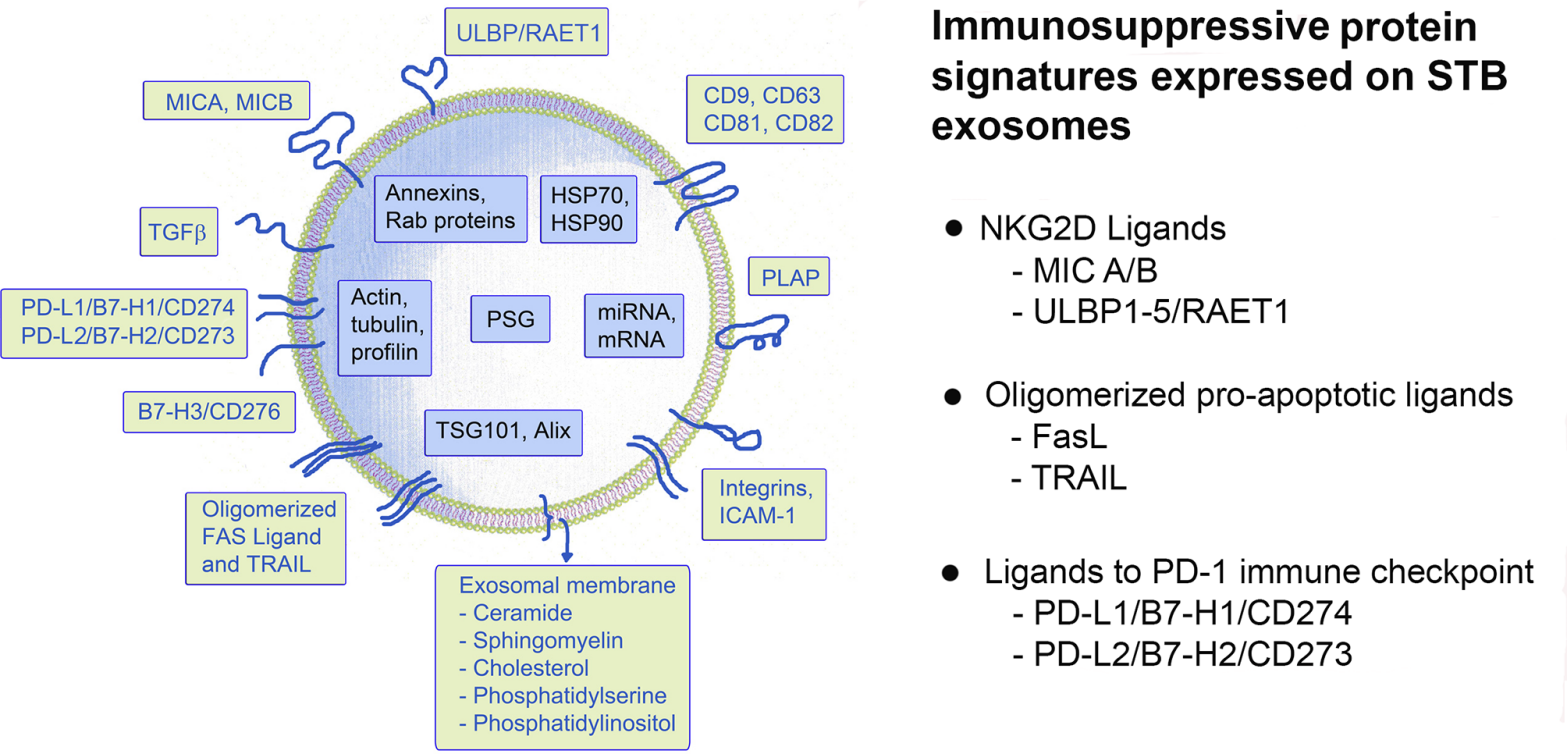
Schematic presentation of a putative STB-derived exosome and a summary of the immunosuppressive signatures, discussed in this review.

The maternal-fetal tolerance is not a uniform event but comprises a “jigsaw puzzle” of molecules and mechanisms that regulate the maternal immune system and ensure the successful implantation, development and growth of the semiallogeneic fetus. STB-derived exosomes secreted throughout pregnancy provide one such mechanism protecting the feto-maternal unit from immune attack. A schematic presentation of the structure of a placental exosome expressing these and other proteins and nucleic acids is shown in **Figure 3**. Three sets of defined signaling protein signatures, carried on STB-exosomes and involved in three major immune pathways are highlighted here. Analyses of these protein/ligand signatures, i.e. (i) MICA/B and ULBP 1-5, ligands of the major activating NK-cell receptor NKG2D; (ii) oligomerized hexamers of FasL and TRAIL, ligands of the Fas- and TRAIL receptors; and (iii) PD-L1 and L2 ligands of the immune checkpoint molecule PD-1, on isolated exosomes can be used for diagnostic purposes to distinguish exosomes with immunoinhibitory function. It is intriguing to realize that placenta protects the fetus by secreting these signaling molecules, ligands to receptors in important basic immune responses, on exosomes. A logical question will be why are these ligands preferably expressed on exosomes and would this be an effective way for modulation of the maternal immune response? Exosomal secretion is a non-classical form of secretion, processed through the endosomal cellular compartment that differs from the “classical” excretory pathway. There are many advantages using exosomes as molecule carriers for intercellular cross-talk and intervention in biological mechanisms: (i) the endosomal processing and anchoring molecules on the exosomal membrane preserve their three dimensional structure and thus their biological activity; (ii) the signaling contact with the receptor is delivered “by proxy”, there is no need for cell-to-cell contact; (iii) the cargo molecules are packaged and concentrated on and in nanometer-sized vesicles which provides higher concentrations of the carried molecules and prevents dilution below the threshold level of activity; (iv) there is good protection of the cargo inside the exosomes due to a very stable exosomal membrane; (v) molecules are recirculated and reused thus there is no dependence of “de-novo” protein synthesis, and last but not least (vi) exosomal secretion comprise an instant delivery of biological effects at a distance (12, 13, 16, 21, 22). Are these advantages important for pregnancy? “Soluble” NKG2D ligands, expressed on the exosomal membrane, besides preserving their tri-dimensional structure and function, seem to be far more potent as receptor down modulators compared to soluble MMP-cleaved ligands (59, 60). The explanation for that is enrichment of the concentration of the same ligand on the exosomal membrane and a probability of expressing several other NKG2D ligands on the same exosome, thus making it a multipotent ligand carrier, impairing the cytotoxic potency of NK and cytotoxic T cells to a greater extent, which will be beneficial to pregnancy. Furthermore, exosomes carrying oligomerized FasL and TRAIL instantly induce apoptosis while FasL and TRAIL expressed on the plasma membrane lose their apoptotic activity and on the contrary, provoke inflammation and promote allograft rejection (41, 48).

**Figure 4:**
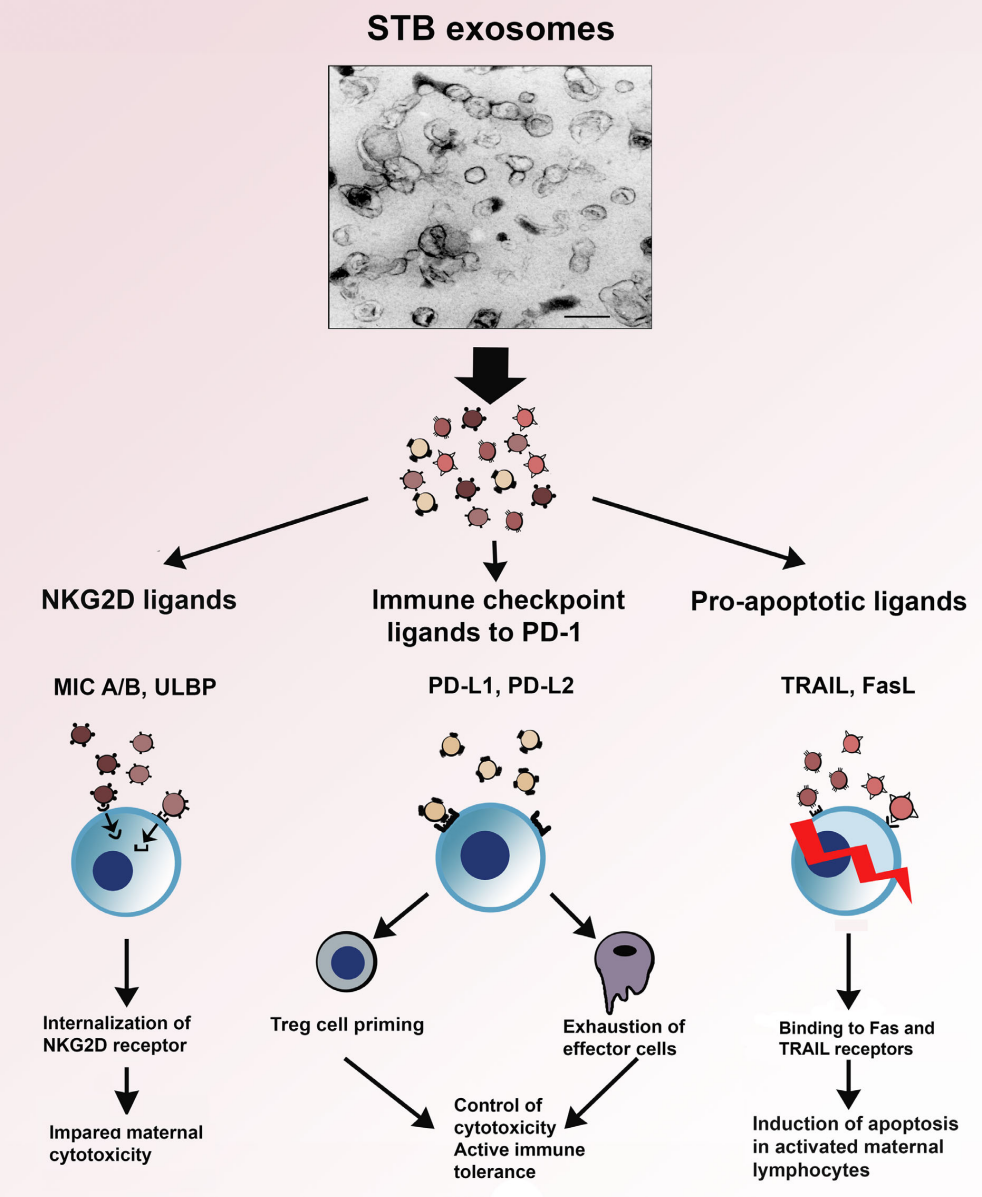
Electron micrograph of STB exosomes isolated from supernatants of placental explant cultures and schematic presentation of their role in three immune mechanisms – NKG2D-mediated cytotoxicity, apoptosis and immune checkpoint regulation.

In **Figure 4**, the interaction of the immunoinhibitory protein signatures on STB exosomes with cytotoxicity, apoptosis and immune checkpoint regulation, is illustrated. As can be seen, the NKG2D-mediated cytotoxicity is downregulated by internalization of the NKG2D receptor; activated immune cells expressing Fas- or TRAIL receptors are eliminated by apoptosis and exosomal PD-L1 and 2 interaction with PD-1 leads to suppression of effector cells and priming of Treg. Common for these protein signatures is that they facilitate immune escape and are not only found on STB exosomes but also carried by exosomes, produced by the vast majority of cancer cells. Pregnancy and cancer do have a similar goal - immune escape of the fetus from the immune system of the mother and of the cancer from the immune system of the host.

The immune inhibition carried out by STB exosomes is selective: (i) the modulation of cytotoxicity is limited to the internalization of the NKG2D receptor, the cytotoxic machinery of the effector cells remains intact (ii); the induction of apoptosis is directed only against activated Fas- or TRAIL receptor-expressing lymphocytes; and (iii) ligation to the immune check point molecule PD-1 promotes generation of Tregs. The highest concentration of STB exosomes is in the intervillous maternal blood and decreases with an increasing distance from the placenta. The continuous release of STB exosomes creates an exosomal concentration gradient, a shield of exosomes around the feto-placental unit where the protection against maternal immune attack is strongest at the feto-maternal interface, i.e. in the immediate vicinity of the chorionic villi. Since the turnover of exosomes is short, their inhibitory influence on maternal immunity would be “fading away” in the systemic maternal circulation. This could be one of the explanations that although the maternal immunity during pregnancy is down regulated it is not completely blunted. The pregnant woman is “semi-immunocompromised”, i.e. sensitive to infections but still able to mount a modified immune response. This immune compromise is also reflected in the fact that pregnant women are at a higher risk for development of hematological malignancies such as leukemias and lymphomas, and breast cancer during and in connection to the gestational time. The partial impairment of the maternal immune defense during pregnancy is the price paid for the complicated, hemochorial mode of reproduction which is required for provision of huge amount of oxygen and nutrients needed for the development of the highly sophisticated human brain. The focus of this review was on the effect of STB exosomes on three major immune mechanisms amongst a plethora of other exosomal functions, not mentioned here. In conclusion, the constitutive placental production and secretion of STB exosomes, carrying immunosuppressive signatures, provide a well-tuned modulation of the maternal immune response and guard the homeostasis of normal pregnancy.

## Author Contributions

LM-N is the single author that raised the idea, wrote the review and suggested/performed the figures and tables.

## Funding

This work was supported by grants from the National Swedish Research Council/Vetenskapsrådet (18–20–345240311),, the Swedish Cancer Society/Cancerfonden (Can 2018/350; 18 07 17), Central ALF funding and the Faculty of Medicine, Umeå University.

## Conflict of Interest

The author declares that the research was conducted in the absence of any commercial or financial relationships that could be construed as a potential conflict of interest.

## Publisher’s Note

All claims expressed in this article are solely those of the authors and do not necessarily represent those of their affiliated organizations, or those of the publisher, the editors and the reviewers. Any product that may be evaluated in this article, or claim that may be made by its manufacturer, is not guaranteed or endorsed by the publisher.
